# Characteristics and treatment of elemental mercury intoxication: A case series

**DOI:** 10.1002/hsr2.293

**Published:** 2021-06-04

**Authors:** Kelly Johnson‐Arbor, Eshetu Tefera, John Farrell

**Affiliations:** ^1^ Department of Plastic and Reconstructive Surgery MedStar Georgetown University Hospital Washington District of Columbia; ^2^ National Capital Poison Center Washington District of Columbia; ^3^ Department of Biostatistics and Biomedical Informatics MedStar Health Research Institute Hyattsville Maryland; ^4^ South Riding Pediatrics Chantilly Virginia

**Keywords:** mercury, poisoning, pediatrics, heavy metals, toxicology

## Abstract

**Background and aims:**

Elemental mercury toxicity is a rare condition which can be difficult to diagnose due to its nonspecific signs and symptoms. The purpose of this investigation is to describe the presenting characteristics and treatment of adult and pediatric patients with elemental mercury poisoning.

**Methods:**

A retrospective review was performed in six patients with elemental mercury exposure or intoxication who were treated in an outpatient medical toxicology clinic. Clinical signs and symptoms, laboratory assessments, and public health responses were reviewed.

**Results:**

Headache, anorexia, rash, and personality changes were commonly reported symptoms in pediatric patients; the adult patients were asymptomatic or reported signs and symptoms included myalgias, tremors, and hypertension. Delays in diagnosis were common. Symptomatic patients had 24‐hour urine mercury concentrations greater than 20 mcg/L. Treatment, including removal from the exposure source as well as chelation with dimercaptosuccinic acid, resulted in resolution of signs and symptoms within 6 months of diagnosis.

**Conclusion:**

The evaluation and treatment of patients with suspected elemental mercury poisoning frequently require a multidisciplinary approach including medical toxicologists and public health officials. A heightened awareness of the clinical presentations of this condition, as well as early identification and removal of patients from the source of exposure and consideration of chelation therapy, can result in accelerated patient recovery.

## 1 INTRODUCTION

Mercury exists in elemental, inorganic, and organic forms; the toxicity of mercury depends on the form as well as the route and chronicity of exposure. Elemental mercury (“quicksilver”) is a nonessential heavy metal element and is one of few metals that exist as a liquid at room temperature.^1^ Elemental mercury toxicity is a rare condition which can occur after exposure to mercury‐containing devices including thermometers, barometers, batteries, and older sphygmomanometers.^2^ Intoxication may also result from the use of elemental mercury in spiritual practices or rituals, including Santeria and Voodoo, as well as occupational endeavors such as gold mining and extraction.^3^, ^4^ The clinical presentation of elemental mercury intoxication is often nonspecific in nature and may be easily misdiagnosed. The signs and symptoms of elemental mercury intoxication are distinctly different from the clinical effects of other forms of mercury intoxication; inorganic mercury toxicity results in gastrointestinal and neurological symptoms, while organic dimethylmercury exposure is associated with delayed cerebellar damage which is often fatal.^5^, ^6^ As elemental mercury intoxication is rarely encountered in clinical practice, physicians caring for patients with suspected mercury toxicity may be unaware of the clinical characteristics of this disease process as well as the diagnostic methods and available treatments for this condition. The objective of this report is to describe the characteristics and treatment of elemental mercury intoxication in adult and pediatric patients treated in the outpatient medical toxicology clinic at MedStar Georgetown University Hospital from September 2015 through February 2019.

## 2 METHODS

Institutional review board (IRB) approval was obtained from the MedStar IRB system, to conduct a retrospective review of adult and pediatric patients treated for mercury vapor exposure or toxicity in the outpatient medical toxicology clinic at MedStar Georgetown University Hospital from September 2015 to February 2019. Six patients, representing two different exposure cases, were identified. Written informed consent for medical case publication was obtained from all patients; for pediatric patients, informed consent was obtained from the parents.

The first case involved an exposure that affected a family of five individuals. In this case, a previously healthy 4‐year‐old boy developed a constellation of unexplained signs and symptoms, including diaphoresis, headache, tactile sensitivity, unwillingness to ambulate, decreased appetite, insomnia, and nonischemic priapism. Symptoms occurred approximately 1 month after the patient's family moved into a new home. The patient's two sisters (ages 6 and 8 years) developed personality changes and complained of headaches. The family dog also became ill and died unexpectedly. Due to their severe symptoms, the 4‐year‐old boy and his oldest sister were unable to attend school on a full‐time schedule. On physical examination, the boy was irritable and withdrawn; he would not ambulate and was transported in a stroller by his parents. After a comprehensive medical workup was unrevealing, the family's pediatrician ordered a 24‐hour urine assay for heavy metals for the youngest child, which revealed an elevated urine mercury concentration (Table 1). The patient's siblings and parents were then tested and were found to have elevated urine mercury concentrations. The local fire department evaluated the patient's home and detected the presence of elevated mercury vapor concentrations within the residence. The family was evacuated from the home for several weeks, while residential mercury assessment and remediation were conducted. The children were evaluated in an outpatient medical toxicology clinic and were prescribed oral chelation therapy with dimercaptosuccinic acid (DMSA [10 mg/kg every 8 hours for 5 days, followed by 10 mg/kg every 12 hours for 14 days]); their parents, who were asymptomatic and had normal physical examinations, were not offered chelation therapy. The source of the exposure was eventually traced back to a spill of elemental mercury in the home which occurred prior to the patient and his family moving into the residence. The highest concentrations of mercury vapor were identified in the home's basement, adjacent to the heating, ventilation, and air conditioning (HVAC) system. Mercury from the spill had likely entered the HVAC system and traveled through the ductwork to the other residential areas, leading to inhalational mercury vapor exposure for the entire family. The previous homeowner did not provide additional details on the prior use of mercury in the home, and it is unknown if this individual had symptoms related to mercury vapor exposure. Once remediation was completed, the family moved back into the home. The children's symptoms improved over the following several months. Over 3 years later, all family members remained asymptomatic.

**TABLE 1:** Characteristics of family members involved in a residential elemental mercury exposure

Age/Sex	4/M	6/F	8/F	36/M	37/F	40/F
Signs/symptoms	Headache, diaphoresis, anorexia, weight loss, fatigue, insomnia, tactile sensitivity, rash	Headache, extremity pain	Abdominal pain, anorexia, headache, rash, insomnia, irritability	None	None	Extremity pain, night sweats, tremors, hypertension
Initial urine mercury concentration (mcg/L)	52	36	55	19	7	53
Initial whole blood mercury concentration (mcg/L)	12	N/A	N/A	7	N/A	34

The second case involved an adult female who developed proximal muscle pain, dental pain, night sweats, tremors, and hypertension. Her primary care physician referred her for neurological and rheumatological evaluations; her erythrocyte sedimentation rate was found to be elevated, and electromyography demonstrated borderline peripheral neuropathy. Other laboratory assays, including complete blood count and comprehensive metabolic panel, were unremarkable. A urinalysis was positive only for moderate blood. She was diagnosed with fibromyalgia and treated with tramadol, trazodone, meloxicam, and physical therapy. Amlodipine and hydrochlorothiazide were prescribed for hypertension. The patient researched her symptoms online and became concerned that she was affected by heavy metal poisoning. She requested testing from her primary care physician; a 24‐hour urine heavy metal screen revealed an elevated urine mercury level, and a whole blood mercury concentration was also elevated. The patient was referred to an outpatient medical toxicology clinic, where oral chelation with DMSA (10 mg/kg every 8 hours for 5 days, followed by 10 mg/kg every 12 hours for 14 days) was initiated. The state Emergency Response department was contacted and performed mercury vapor assessment of the patient's home. Elevated mercury vapor concentrations were detected on the patient's work clothes; there were no other significant vapor concentrations found in the patient's home. The patient reported that she worked as a home health aide at two different locations including a medical facility and a private home. Both work locations were then tested, but mercury vapor concentrations were not elevated, and no source of mercury exposure was found. Assessment of a previous work location, a private home, revealed elevated mercury vapor concentrations outside of the residence; the patient had stopped working at that location around the time she became ill. The local health department was unable to access the home for further testing, so the source of mercury exposure was never identified. The patient tolerated her chelation therapy without difficulty; her symptoms resolved within several months of her diagnosis, and she was able to discontinue all antihypertensive and analgesic medications within 4 months of her diagnosis.

## 3 RESULTS

Patient demographic information and pertinent laboratory values are presented in Table 1. Serial urine mercury concentrations were obtained from all symptomatic patients, and serial blood mercury concentrations were obtained from the symptomatic adult patient.

Elimination half‐lives were calculated by the nonlinear least‐squares regression method (uncorrected for renal function). The elimination half‐life of mercury in the urine ranged from 1.9‐6.0 months (mean 4.8 months) in the patients who received chelation therapy. Serial urine mercury concentrations of the two patients with the highest exposures (initial urine mercury concentration > 50 mcg/L) are depicted in Table 2. The half‐life of mercury in the blood was 2.7 months. Both urine and whole blood mercury concentrations decreased once patients were removed from the source of mercury exposure and treated with chelation therapy. Due to a desire to become pregnant, one patient was followed in the medical toxicology clinic until both blood and urine mercury concentrations were undetectable. In this patient, whole blood mercury concentrations were detectable (>0 mcg/L) for 8 months after the diagnosis was established, and urine mercury concentrations were detectable (>0 mcg/L) for 1 year after diagnosis.

**TABLE 2:**
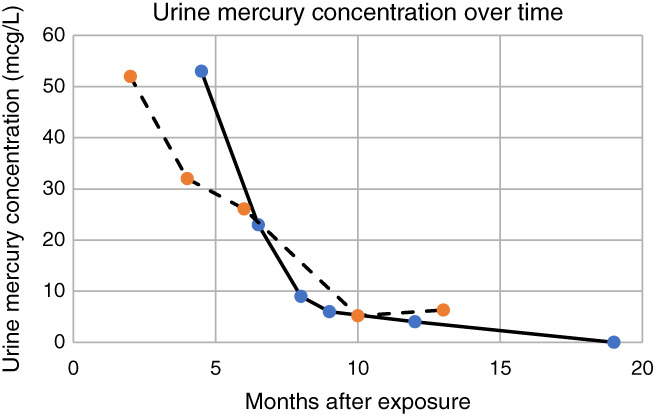
Relationship of urine mercury concentration vs time

**FIGURE 1:**
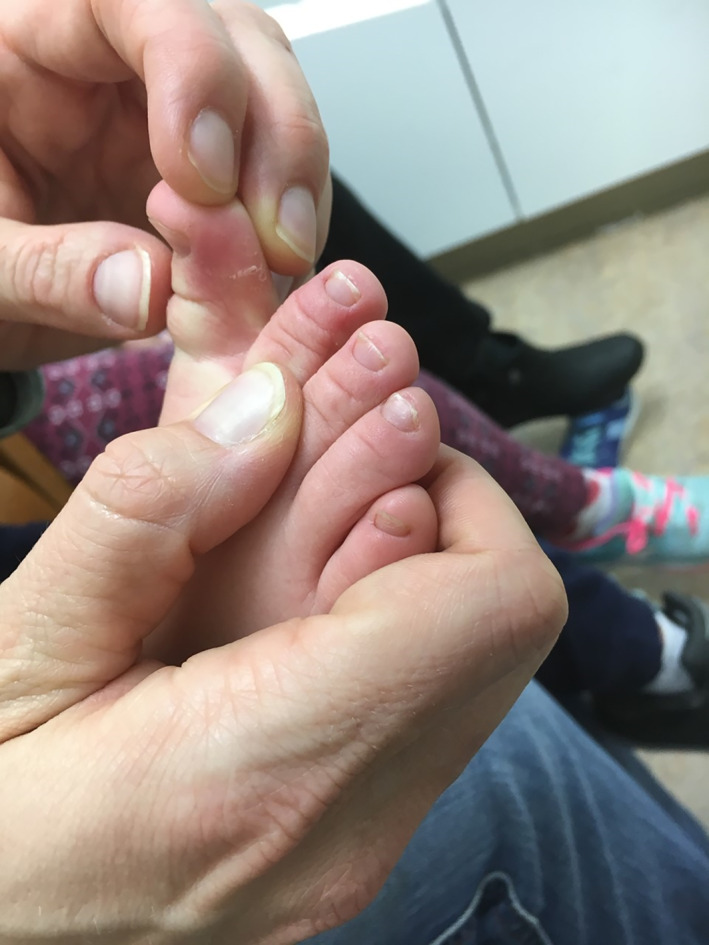
Acrodynia

Signs and symptoms of mercury vapor intoxication were noted in patients with 24‐hour urine mercury concentrations greater than 20 mcg/L. Commonly reported signs and symptoms in the pediatric patients included headache (3/3 patients), anorexia (2/3), rash (2/3), and personality changes including irritability (2/3). Pink discoloration and desquamation of the distal extremities, consistent with acrodynia, was noted in the youngest patient (Figure 1). The signs and symptoms of mercury intoxication improved within a month of chelation therapy initiation and exposure source removal; within 6 months, all patients had complete resolution of their signs and symptoms.

Symptomatic patients were treated with oral chelation therapy; a single course of DMSA (10 mg/kg orally every 8 hours for 5 days, followed by 10 mg/kg orally every 12 hours for 14 days) was prescribed. As signs and symptoms improved rapidly after the removal from the source of exposure and a single course of chelation therapy, additional courses of DMSA were not administered. Chelation therapy was tolerated well by all subjects; although hepatic transaminase elevation has been reported as a consequence of DMSA therapy, no abnormalities in hepatic transaminases were noted in the patients in this series.

## 4 DISCUSSION

Elemental mercury is a dense, silvery‐colored metal that exists in a liquid state at room temperature. It is 13 times denser than water; due to this, estimation of spill severity may be misleading as small volumes may correspond with large amounts of the compound.^7^ A “small” mercury spill is often defined as the equal to or less than the amount of mercury in a fever thermometer (generally <0.7 g).^7^ While small mercury spills are generally not associated with elevated mercury vapor concentrations, the vapor can easily accumulate in small, poorly ventilated, or low‐lying areas, increasing the potential for toxicity.^7^, ^8^ The low vapor pressure of elemental mercury results in easy and rapid volatilization at room temperatures; as mercury vapor is odorless and tasteless, its presence can be difficult to detect without specialized equipment.^9^, ^10^

When spilled, elemental mercury forms small beads which spread easily and are difficult to clean up. Attempts to decontaminate elemental mercury beads by the use of a traditional home vacuum cleaner may enhance the volatility and spread. Elemental mercury also tends to soak into porous materials and may be difficult to remove from carpeting, clothing, unfinished wood, and upholstered furniture; contaminated portions of porous surfaces may be cut out, removed, and disposed of in accordance with local, state, or federal regulations.^7^ For small spills, mercury removal from hard surfaces can be achieved by using cardboard, masking tape, and eyedroppers; commercially available powdered sulfur may also be used to adsorb elemental mercury from hard surfaces.^7^, ^8^ Larger mercury spills require comprehensive evaluation and specialized remediation.

Inhalational exposure represents the primary source of toxicity after elemental mercury exposure. Ingestion does not result in significant exposure as elemental mercury is poorly absorbed from the gastrointestinal tract, and dermal penetration is also limited.^11^, ^12^ Approximately 80% of an inhaled dose of elemental mercury is absorbed by the lungs and systemically distributed throughout the circulation to all organs. Absorbed elemental mercury is oxidized in vivo to form inorganic mercurous (Hg^+^) and mercuric (Hg^+2^) ions; these ions bind with sulfhydryl groups, leading to inactivation of enzymes and altered cell membrane permeability.^13^ The proximal convoluted tubule is the primary site of deposition of inorganic mercury, and renal excretion predominates.^14^ Elemental mercury vapor exposure can affect multiple organ systems, with the brain and kidneys primarily affected.^15^ Elemental mercury crosses the blood–brain and placental barriers readily.^16^ Children are more susceptible than adults to the toxic effects of mercury vapor, as their short stature places them in closer proximity to the ground where dense mercury vapors settle.^17^ In addition, children have a higher minute ventilation than adults, leading to increased inhalation of mercury vapors.^18^

The signs and symptoms of mercury vapor intoxication can be subtle and nonspecific in nature and may include weakness, pain, anorexia, weight loss, and gastrointestinal or neurologic symptoms.^14^ After an acute high‐level exposure to mercury vapor, pneumonitis characterized by fevers, cough, and dyspnea may occur with hours and may result in mortality due to progressive hypoxia.^19^, ^20^ In children, the most common presenting symptom of mercury poisoning is headache; this was a significant presenting symptom in each of the children in this case series.^20^ Mercury interferes with catecholamine breakdown by inactivation of catecholamine‐*O*‐methyltransferase, leading to accumulation of catecholamines and development of hypertension, diaphoresis, and tachycardia in some patients.^21^ Acrodynia or “pink disease,” a syndrome characterized by painful pink discoloration and peeling of the hands and feet, is occasionally noted in young children exposed to elemental mercury; the etiology of acrodynia may be related to an underlying hypersensitivity to mercury.^22^ Mercurial erethism, which was noted in several patients in this series, is characterized by personality changes including irritability, insomnia, and shyness.^23^

**FIGURE 2:**
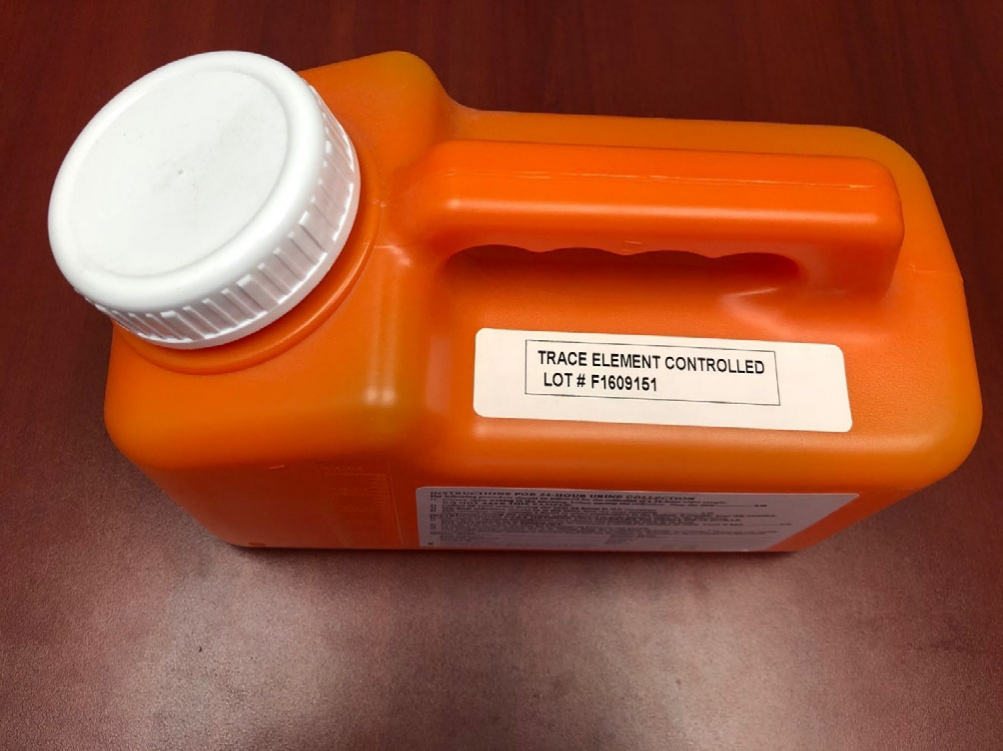
A trace metal‐free 24‐hour urine collection container

Given the renal excretion pattern of elemental mercury, urine is the preferred method of diagnosis for most elemental mercury exposures. Urine is also the best marker of exposure for most inorganic mercury exposures; as organic mercury is excreted through the bile and feces, it is not readily detectable in the urine.^5^, ^24^ As the half‐life of elemental mercury is longer in the urine than in whole blood, urine mercury concentrations are the best indicator of long‐term exposure to elemental mercury while whole blood concentrations are more useful for short‐term, high‐level exposures.^15^ A 24‐hour urine assay is the standard test for elemental mercury exposure; ideally, this should be collected in a trace metal‐free collection container to reduce potential cross‐contamination (Figure 2).^25^ The 24‐hour urine mercury level may not always correlate with the signs and symptoms of toxicity; this may be due to variations in the timing and degree of exposure in relation to the timing of testing.^26^

Due to the nonspecific signs and symptoms associated with this illness, diagnosis of mercury poisoning may be difficult to establish. A comprehensive evaluation of environmental and occupational exposure sources may help establish the diagnosis. Patients with elemental mercury intoxication may be misdiagnosed by health care providers as having a viral‐type illness or rheumatologic disorder.^27^, ^28^, ^29^ A missed or delayed diagnosis occurred in all symptomatic patients in this case series. In one patient, the presenting signs and symptoms of elemental mercury toxicity were initially diagnosed as fibromyalgia; the diagnosis of this patient, an underrepresented minority, may have been affected by implicit biases of the healthcare providers. Although the topic of implicit bias is rarely discussed within the context of accidental intoxications, these unconscious biases may contribute to misdiagnosis and delays to definitive care in vulnerable poisoned populations.

Elemental mercury exposures often represent public health dangers and may require involvement of local, regional, or national authorities for assessment and remediation. The geographical locations of mercury exposure in this case series involved three separate states. The public health response varied between jurisdictions; depending on the location, mercury vapor assessment was provided by the local health department, state Emergency Response team, or local fire department. While state and regional health departments are useful resources for the initial assessment of recreational or elemental mercury exposures, their availability, resources, and engagement can vary based on location.^30^ In the United States, the Environmental Protection Agency (EPA) can respond to larger elemental mercury spills that represent an imminent and substantial endangerment to public health.^31^ Elemental mercury spills of more than one pound or 453 g (the equivalent of two tablespoons or 30 mL) must be reported to the EPA's National Response Center (NRC) at phone number 800‐424‐8802. The NRC hotline is available 24 hours a day, 7 days a week.^32^ After the source of mercury exposure is identified, remediation may be indicated. Remediation should be performed by experienced contractors. The remediation process may be lengthy; for residential exposures, temporary relocation of home dwellers may be required.

Identification and elimination of the source of mercury exposure is of paramount importance for the treatment of patients with elemental mercury toxicity. In addition, chelation therapy is often utilized for patients with mercury poisoning who are symptomatic; however, the clinical benefit of chelation therapy in symptomatic patients is poorly defined, and this treatment is of lesser significance than identification and removal of the exposure source.^33^ Chelation agents form chemically inert and nontoxic complexes with metal ions, which are then excreted.^34^ Since chelation therapy releases metals from body tissues, urine mercury concentrations may transiently increase upon initiation of chelation therapy before significant decreases occur.^21^ Chelators used in the treatment of mercury intoxication may include British anti‐Lewisite (BAL), dimercaptosuccinic acid (DMSA, succimer), and 2,3‐dimercaptopropane sulfonic acid (DMPS, unithiol). In the United States, DMSA is a commercially available oral chelator; although it is only approved by the United States Food and Drug Administration for treatment of lead toxicity, it can also be utilized for patients with mercury poisoning. Since heavy metal poisoning is an uncommon diagnosis in the United States, DMSA may not be readily available in pharmacies, and pharmacy stock may be limited. The challenges in locating and obtaining chelators such as DMSA may be related to increases in prescription drug shortages in the United States. These drug shortages, which often affect the supply of orphan drugs used to treat rare diseases, have increased in recent years and represent a significant public health concern in the United States.^35^ In this case series, DMSA was able to be obtained from local chain pharmacies. The pharmacies did not carry it in their normal stock and had to order it as a “drop ship” from the manufacturer; this resulted in a 1‐ to 2‐week delay in delivery to the patients. Once obtained, the patients in this series tolerated DMSA chelation without significant complications. Common adverse effects associated with DMSA administration include bad taste and smell, mild gastrointestinal complaints, elevation of hepatic transaminases, and increased excretion of trace elements such as zinc and copper.^36^ Overall, DMSA is well tolerated by most individuals; for the youngest children in this series, palatability of DMSA was enhanced by opening the capsules and mixing the contents with chocolate frosting, applesauce, or pudding.

DMPS may also be considered as a chelating agent for mercury intoxication. DMPS, a water soluble analogue of BAL, is produced and marketed by the German pharmaceutical company Heyl, Berlin; it is approved in Germany for oral and intravenous treatment of acute heavy metal intoxications.^37^ Although DMPS is not currently approved by the United States Food and Drug Administration for use in chelation therapy, it can be obtained through compounding pharmacies in oral or intravenous formulations.^38^ DMPS has been successfully used in the treatment of mercury intoxication.^39^ Compared with DMSA, DMPS remains in the body for longer, acts more quickly, and is more effective in chelating patients with inorganic mercury intoxication.^40^ DMPS is generally well tolerated; it can cause a dose‐dependent increase in urinary copper excretion, and Stevens‐Johnson syndrome has been reported to occur after administration of DMPS.^41^, ^42^

The patients described in this case series all had a favorable clinical outcome once the diagnosis of elemental mercury intoxication was established, suggesting that the significant clinical effects that may occur as a consequence of this intoxication are reversible with treatment including source identification and remediation as well as chelation therapy. A significant limitation of this analysis is the low number of patients included in this case series. As elemental mercury intoxication remains a rare diagnosis, most published descriptions of affected patients involve case reports or small case series.

## 5 CONCLUSION

Although rarely encountered in medical practice, elemental mercury intoxication represents a public health concern that can be associated with significant morbidity in affected individuals. The signs and symptoms of mercury poisoning are often attributed to other disease processes, leading to diagnostic and treatment delays. The assessment, diagnosis, and treatment of elemental mercury intoxication often require a team approach with involvement of poison centers and public health authorities. Adequate identification and removal of the exposure source as well as use of chelation therapy for symptomatic individuals can result in complete resolution of the signs of symptoms of mercury intoxication. An enhanced understanding of the clinical presentations of elemental mercury poisoning may allow health care providers to have a heightened understanding of this condition, leading to decreased misdiagnosis due to implicit biases and increased awareness of the potential for mercury intoxication in patients who present with nonspecific signs and symptoms which cannot be attributed to another likely diagnosis.

## CONFLICT OF INTEREST

The authors have no conflicts of interest to disclose.

## AUTHOR CONTRIBUTION

Conceptualization: Kelly Johnson‐Arbor

Resources: John Farrell

Formal analysis: Eshetu Tefera

Writing‐ original draft: Kelly Johnson‐Arbor, John Farrell

Writing‐ review and editing: Kelly Johnson‐Arbor, John Farrell, Eshetu Tefera

  All authors have read and approved the final version of the manuscript. Kelly Johnson‐Arbor had full access to all of the data in this study and takes complete responsibility for the integrity of the data and the accuracy of the data analysis.

  Kelly Johnson‐Arbor affirms that this manuscript is an honest, accurate, and transparent account of the study being reported; that no important aspects of the study have been omitted; and that any discrepancies from the study as planned have been explained.

## INSTITUTION AND ETHICS APPROVAL AND INFORMED CONSENT

The work was performed at MedStar Georgetown University Hospital and was approved by the Georgetown University Institutional Review Board. Written informed consent for publication was also obtained from each patient described in the manuscript.

## TRANSPARENCY STATEMENT

This manuscript is an honest, accurate, and transparent account of the study being reported. No important aspects of the study have been omitted; any discrepancies from the study as planned (and, if relevant, registered) have been explained.

## Data Availability

The data that support the findings of this study are available from the corresponding author upon reasonable request.
